# Supplementary figures and images for: Genetic and Functional Differences between Duplicated Zebrafish Genes for Human SCN1A
Source: Cells. 2022 Jan 28;11(3):454. doi: 10.3390/cells11030454 (PMC8834172; doi:10.3390/cells11030454)

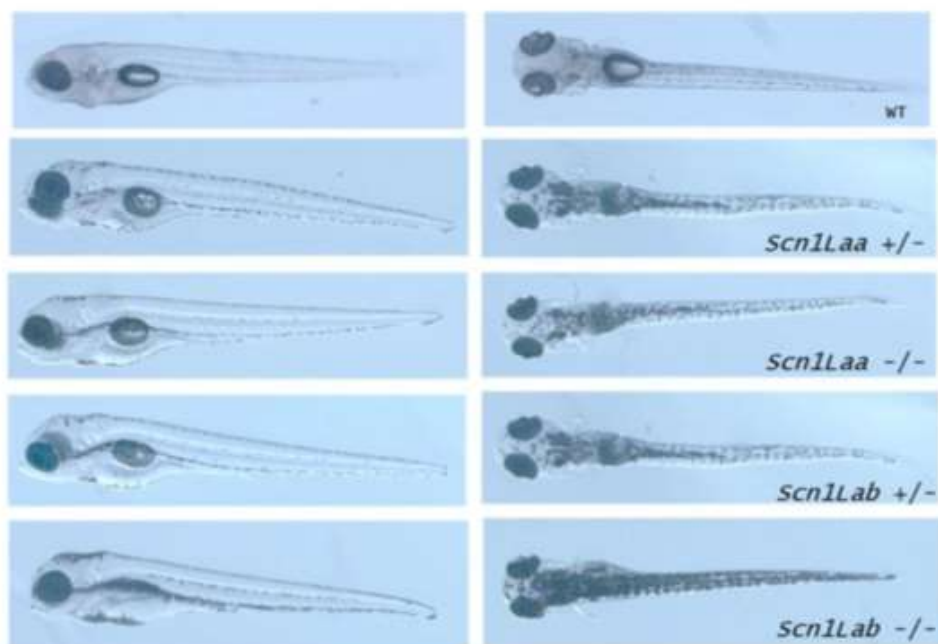

Figure S1: Morphology & strain comparison

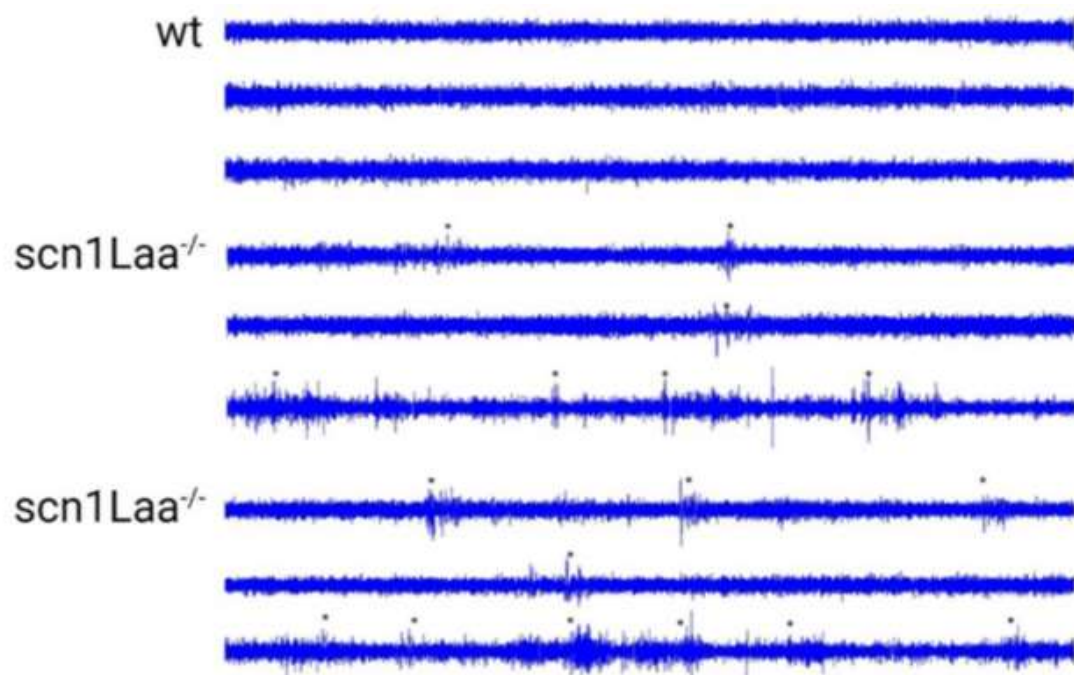

Figure S2: Full-length LFP recordings.

Supplement: Supplementary file 1 [file cells-11-00454-s001.zip › cells-1557106-supplementary.pdf]
